# Effects of Extraction Processes on the Oxidative Stability, Bioactive Phytochemicals, and Antioxidant Activity of Crude Rice Bran Oil

**DOI:** 10.3390/foods11081143

**Published:** 2022-04-15

**Authors:** Tiraporn Junyusen, Natthaporn Chatchavanthatri, Pansa Liplap, Payungsak Junyusen, Van Man Phan, Siriwan Nawong

**Affiliations:** 1 School of Agricultural Engineering, Institute of Engineering, Suranaree University of Technology, Nakhon Ratchasima 30000, Thailand; chat.natthaporn@gmail.com (N.C.); pansa@sut.ac.th (P.L.); payungs@sut.ac.th (P.J.); nguyenphan05031987@gmail.com (V.M.P.); 2 Synchrotron Light Research Institute (Public Organization), Nakhon Ratchasima 30000, Thailand; siriwannawong@slri.or.th

**Keywords:** crude rice bran oil, thermal cooking pretreatment, ultrasonic pretreatment, oxidative stability, bioactive compounds, antioxidant activity

## Abstract

This research investigates the effects of different extraction processes on the oil extractability, oxidative stability, bioactive compounds, and antioxidant activity of crude rice bran oil (CRBO). The experimental extraction processes include hexane extraction (HE), cold press extraction (CE), thermally pretreated cold press extraction (CCE), and ultrasound-pretreated cold press extraction (UCE). The results show that thermal cooking and ultrasound pretreatment significantly improve the oil extractability of the cold press extraction process. The oil yields of CE, CCE, and UCE were 14.27, 17.31, and 16.68 g oil/100 g rice bran, respectively. The oxidative stability of CE and CCE oils was higher than HE and UCE oils, as evidenced by the synchrotron-radiation-based Fourier transform infrared (SR-FTIR) absorption peak. The ρ-anisidine values of HE, CE, CCE, and UCE were 0.30, 0.20, 0.91, and 0.31, respectively. Meanwhile, ultrasound pretreatment significantly reduced the bioactive compounds and chemical antioxidant activity of UCE oil. The CE, CCE, and UCE oils (0.1% oil concentration) exhibited higher inhibitory effects against hydrogen-peroxide-induced cellular oxidative stress, compared to HE oil (0.39% oil concentration). Essentially, CCE is operationally and environmentally suitable for improving the oil yield, oxidative stability, bioactive compounds, and antioxidant activities of CRBO.

## 1. Introduction

A by-product of rice processing, rice bran contains about 15–23% oil and thus is a good source for rice bran oil (RBO). RBO is rich in unsaturated fatty acids, including oleic acid, linoleic acid, and linolenic acid [1]. RBO is also a rich source of bioactive phytochemicals such as γ-oryzanol, tocols (α/γ-tocopherol and α/γ-tocotrienol), phenolic compounds, flavonoids, squalene, and phytosterols [2,3]. The bioactive phytochemicals possess high antioxidant capacities against free radicals [4,5], in addition to various health benefits, such as lowering serum cholesterol, platelet aggregation, and cholesterol absorption [4,6]. As a result, RBO is widely used in food, cosmetic, and pharmaceutical products [1,4].

The chemical antioxidant assays commonly used to assess the in vitro antioxidant activity of oil extracts include 2,2-diphenyl-1-picrylhydrazyl (DPPH), 2,2-azinobis (3-ethyl-benzothiazoline-6-sulfonic acid) (ABTS), and ferric reducing antioxidant power (FRAP) [2,7]. The advantages of DPPH, ABTS, and FRAP assays are ease of use, low cost, and reproducibility [8,9]. Meanwhile, the cellular antioxidant activity assay measures the in vivo antioxidant activity. Specifically, it evaluates the uptake, distribution, bioavailability, and metabolism of compounds in the cells [10,11]. Lu et al. [7], Liu et al. [2], and Yuwang et al. [12] assessed the antioxidant activity of RBO and rice bran extracts using the chemical- and cellular-based assays.

The oxidative stability of oils is closely related to their fatty acid compositions and bioactive phytochemicals with antioxidant capacities. The bioactive phytochemicals stabilize unsaturated fatty acids and provide protection against oxidative stress [6,13,14]. The oxidative stability plays an essential part in the shelf life, quality, and nutritional value of oils [15]. In other words, the oxidative stability of oils is positively correlated with the shelf life, quality, and nutritional value of oils.

Free fatty acid (FFA), peroxide value (PV), and ρ-anisidine value (ρ-AV) have been used to assess the oxidative stability of oils [15,16]. Meanwhile, spectroscopic analysis in the infrared range measures the vibration in the chemical bonds of functional groups [17]. Previous research utilized spectroscopic methods, such as Fourier transform infrared spectroscopy (FTIR), to determine the properties, quality, and oxidative stability of vegetable oils. Specifically, FTIR was used to determine the free fatty acid [18] and ρ-anisidine value (ρ-AV) [15] and to characterize the oxidation process of oil [19] and adulteration of vegetable oils [20].

Extraction processes play an important role in the oil extractability, quality, and phytochemicals of crude rice bran oil (CRBO). Existing CRBO extraction techniques include cold press extraction (CE) [5], solvent extraction (SE) [5], supercritical fluid extraction (SFE) [21,22], microwave-assisted extraction (MAE) [23], and aqueous enzymatic extraction (AEE) [1].

CE, SE, and pre-pressed SE are commonly used in industrial extraction of RBO. The advantages of CE include its high content of natural antioxidants, low cost, and ease of implementation. However, the oil extractability is low. In comparison, the oil extractability of SE and pre-pressed SE are higher but both methods are time-consuming and chemically toxic [22,24,25,26]. Alternatively, thermally pretreated (thermal cooking) cold press extraction (CCE) and ultrasound-pretreated cold press extraction (UCE) are efficient extraction methods of RBO [24,25]. In addition, both CCE and UCE are more straightforward, economical, and efficient compared to the conventional extraction techniques (i.e., CE, SE, and pre-pressed SE). According to Ali et al. [6], Trevisani Juchen et al. [22], Phan et al. [27], Liu et al. [2], and Xu et al. [1], the extraction processes of vegetable oils directly affect the oil extractability, oxidative stability, bioactive phytochemicals, and antioxidant activity.

As a result, this research aims to comparatively investigate the oil extractability, oxidative stability, bioactive compounds, and chemical and cellular antioxidant activities of CRBO from different extraction processes. The experimental extraction processes include hexane extraction (HE), cold press extraction (CE), thermally pretreated (thermal cooking) cold press extraction (CCE), and ultrasound-pretreated cold press extraction (UCE). In addition, synchrotron-radiation-based Fourier transform infrared spectroscopy (SR-FTIR) was performed to characterize the oxidative stability of CRBO. The findings are expected to shed light on the extraction process that is operationally and environmentally suitable for the production of high-quality CRBO.

## 2. Materials and Methods

### 2.1. Raw Materials

In this research, rice bran (RB) of the jasmine variety was purchased from Korat Yongsanguan Rice mill Co., Ltd. (Nakhon Ratchasima, Thailand). Prior to the experiment, the RB was sieved by a 60-mesh sieve (0.25 mm) and oven-dried (Universal Oven UF 110, Memmert GmbH + Co. KG; Schwbach, Germany) at 100 °C for 15 min to inactivate endogenous lipase [24]. The moisture content of dried RB was 6–8%, and it was vacuum-packed and retained at 4 °C for subsequent analysis. All chemicals used were of analytical grade.

### 2.2. Extraction of Crude Rice Bran Oil (CRBO)

The CRBO extraction was performed on a laboratory scale following the method by Phan et al. [24] with minor modifications. Specifically, CRBO was extracted using four extraction processes: HE, CE, CCE, and UCE. In HE, the extraction was carried out at 90 °C for 4 h, with an RB to solvent ratio of 1:10. The solvent was evaporated, and the CBRO was collected. In CE, the extraction was performed at 40 ± 2 °C using a cold press machine (Dulong, DL-ZY J02; Nanchang, China).

In CCE, RB was thermally pretreated (i.e., thermal cooking) at 100 °C for 5 min under a moist-heat condition using an autoclave (Tommy, SX700; Tokyo, Japan). The thermally pretreated RB was oven-dried at 100 °C for 25–30 min to reduce the moisture content to 6–8% before cold press extraction at 40 ± 2 °C.

In UCE, RB was first mixed in distilled water at a ratio of 1:3 and pretreated using an ultrasound processor (VCX750 Vibracell, Sonic & Materials, Inc.; Newtown, CT, USA) at 4.50 W/g ultrasonic power and 10 min irradiation time with pulse durations of 50 s on and 10 s off at 30 °C. The ultrasound-pretreated RB was filtrated and oven-dried at 100 °C for 25–30 min to reduce the moisture content to 6–8% before cold press extraction at 40 ± 2 °C. The CRBO from the HE, CE, CCE, and UCE processes was retained at 4 °C for subsequent analysis. The CRBO yield was calculated by dividing the weight of extracted CRBO by the initial weight of RB, as shown in Equation (1).

CRBO yield (%) = W_1_/W_2_ × 100 (1)

where W_1_ is the weight of extracted oil (g) and W_2_ is the initial weight of the RB (g) used in each extraction.

### 2.3. Physicochemical Analysis

The physicochemical analysis followed the American Oil Chemists’ Society (AOCS) official methods [28]: Ca 5a-40 for free fatty acid (FFA), Cd 8b-90 for peroxide value (PV), and Cd 18–90 for ρ-anisidine value (ρ-AV). Total oxidation value (TOTOX) was calculated by: TOTOX = (2 PV) + (ρ-AV). The CRBO color was measured by a Lovibond tintometer (PFXi-880/L, Tintometer Ltd.; Salisbury, UK) in a 1-inch glass cell in transmittance mode and expressed as red (R), yellow (Y), and 5R + Y (i.e., Lovibond color unit). The experiments were carried out in triplicate.

### 2.4. Determination of Total Phenolic Content (TPC)

Total phenolic content (TPC) was determined in accordance with the method in Trevisani Juchen et al. [22] with minor modifications. Prior to the measurement, 50 mg of CRBO was mixed with 1 mL methanol. The mixture (0.5 mL) was then mixed with 2.5 mL of Folin–Ciocalteu reagent and shaken well for 6 min before the addition of 2 mL of 10% (*w*/*v*) sodium carbonate and incubated at room temperature in the dark for 2 h. The absorbance of supernatant was taken at 765 nm using a UV-spectrophotometer/NIR (Shimazu, UV-2600; Kyoto, Japan). The TPC results were expressed as milligram of gallic acid equivalent per g of CRBO (mg GAE/g). The experiments were carried out in triplicate.

### 2.5. Determination of Total Flavonoid Content (TFC)

The measurement of total flavonoid content (TFC) followed the method by Jun et al. [29] with minor modifications. Prior to the analysis, 0.5 mL of CRBO was dissolved in 4.5 mL methanol and then mixed in 1.25 mL distilled water and 75 µL of 5% NaNO_2_. The mixture was incubated at room temperature for 6 min before adding 150 µL of 10% AlCl_3_ solution. The mixture was retained in the dark for 5 min before adding 0.5 mL of 1.0 M NaOH. The solution was shaken vigorously and measured using a UV-spectrophotometer/NIR (Shimazu, UV-2600; Kyoto, Japan) at 510 nm. Catechin was used as standard, and the results were expressed as mg catechin equivalent per g of CRBO (mg CE/g). The experiments were carried out in triplicate.

### 2.6. Determination of γ-Oryzanol Content

The γ-oryzanol content was analyzed using high performance liquid chromatography (HPLC; Agilent 1200 series, Agilent Technologies, Inc.; Santa Clara, CA, USA) equipped with Poroshell 120 EC-C18 column (3.0 mm × 150 mm, 2.7 µm) and diode-array UV/VIS detector (DAD), following the method by Phan et al. [30]. The column temperature and UV detector wavelength were 25 °C and 325 nm. The mobile phase was of gradient binary phase consisting of methanol and acetonitrile of 100:0, 50:50, and 40:60 (*v*/*v*) for 5, 10, and 30 min, respectively. The constant flow rate was maintained at 1.0 mL/min, and the injection volume was 20 µL. The experiments were carried out in triplicate.

### 2.7. Determination of α-Tocopherol Content

The α-tocopherol content was analyzed by HPLC (1260 Infinity II LC system, Agilent Technologies, Inc.; Santa Clara, CA, USA) equipped with autosampler (G1329B), quaternary pump (G1311B), Hypersil ODS column (4.0 × 250 mm, 5 µm, Agilent), and fluorimetric detector (G1321B) at 25 °C and an injection volume of 20 µL, following Chatchavanthatri et al. [31]. The fluorimetric detection was carried out at the excitation and emission wavelengths of 290 and 330 nm. The mobile phase consisted of 50:40:10 (A) and 30:65:5 (B) acetonitrile–methanol–isopropanol mixtures (*v*/*v*/*v*), given the flow rate of 1.0 mL/min. Phase A was isocratically eluted for 5 min, followed by a 10 min gradient from phase A to 100% phase B, with a final 5 min isocratic elution with phase B. The experiments were carried out in triplicate.

### 2.8. Gas Chromatography–Mass Spectrometry (GC–MS)

The squalene, phytosterols, and triterpenoid in the CRBO were separated and identified using GC–MS (Bruker 320 GC/MS System, Bruker Corporation; Billerica, MA, USA). In the analysis, 400 mg of CRBO was first dissolved in 1 mL of dichloromethane and filtered with a 0.45 µm filter. The supernatant (2 µL) was then injected into the GC–MS and separated by an Rtx-5MS capillary column (30 m × 0.25 mm, 0.25 µm fused silica). The initial column temperature was 40 °C and held for 2 min before being increased to 200 °C at a rate of 8 °C/min and then to 280 °C at a rate of 5 °C/min and held for 30 min. The injection temperature was 250 °C. Helium was used as carrier gas in the mobile phase with a flow rate of 1 mL/min and a split ratio of 1:5. The ion source and transmission line temperatures were 240 °C. The ionization mode was electron ionization (EI) with a scan range of 35–550 m/z. The squalene, phytosterols, and triterpenoid were identified by comparing with the standards of the National Institute of Standards and Technology. The results were expressed as a percentage of bioactive phytochemical in CRBO.

### 2.9. Determination of Chemical Antioxidant Capacity

The measurement of DPPH radical scavenging activity followed Chatchavanthatri et al. [31] and Trevisani Juchen et al. [22] with minor modifications. Specifically, the radical solution was first prepared by dissolving 2.4 mg DPPH in 100 mL methanol, and 0.1 mL of CRBO was then added to 2.9 mL of 60 µM DPPH solution. The mixture was shaken and retained in the dark at room temperature for 30 min. The absorbance was measured against the blank at 515 nm using a UV-spectrophotometer (Shimazu, UV-2600; Kyoto, Japan). Trolox was used as standard. The results were reported in mg Trolox equivalent antioxidant capacity per g of CRBO (mg TEAC/g). The experiments were carried out in triplicate.

The measurement of ABTS radical scavenging activity followed the process performed by Chatchavanthatri et al. [31]. The ABTS solution was prepared by reacting 7 mM ABTS with 2.45 mM potassium persulphate and incubated at room temperature in the dark for 16 h. Prior to use, the ABTS reagent was diluted with methanol for absorbance of 0.70 ± 0.02 at 734 nm. In the analysis, 0.1 mL of CRBO and standard solutions (Trolox of 0, 5, 10, 25, 50, and 100 mg/L) were mixed with 3.9 mL ABTS solution and left at room temperature in the dark for 5 min. The absorbance at 734 nm was measured using a UV-spectrophotometer (Shimazu, UV-2600; Kyoto, Japan). The results were expressed in mg Trolox equivalent antioxidant capacity per g of CRBO (mg TEAC/g). The experiments were carried out in triplicate.

The ferric reducing antioxidant power (FRAP) assay is based on the reduction of ferric tripyridyltriazine (Fe^3+^-TPTZ) complex (colorless) to Fe^2+^-TPTZ (blue color) induced by the action of electron-donating antioxidants at low pH. The FRAP antioxidant activity of CRBO was measured spectrophotometrically following the method by Oh et al. [8]. Specifically, the FRAP solution was prepared by mixing 300 mM acetate buffer (pH 3.6), 10 mM TPTZ in 40 mM HCl, and 20 mM FeCl_3_.6H_2_O (10:1:1 (*v*/*v*)). In the analysis, 10 µL of CRBO was mixed with 1.3 mL FRAP solution and incubated at 37 °C in the dark for 4 min. The absorbance was measured against the blank at 593 nm using a UV-spectrophotometer (Shimazu, UV-2600; Kyoto, Japan). Trolox was used as standard. The results were reported in mg Trolox equivalent antioxidant capacity per g of CRBO (mg TEAC/g). The experiments were carried out in triplicate.

### 2.10. Determination of Cellular Antioxidant Capacity

#### 2.10.1. Cell Viability Assessment

The cytotoxicity of CRBO to human hepatocellular carcinoma (HepG2) cells was evaluated by the 3-(4,5-dimethylthiazol-2-yl)-2,5-diphenyltetrazolium bromide (MTT) assay, following the experiment by Hamzeh et al. [10] with some modifications. HepG2 cells were seeded at a density of 7 × 10^3^ cells/well in a 96-well microplate containing 100 µL culture medium (Dulbecco’s modified Eagle’s medium, DMEM) supplemented with 10% (*v*/*v*) fetal bovine serum (FBS) and 1% (*v*/*v*) penicillin–streptomycin. The cells were incubated with 5% CO_2_ at 37 °C for 24 h. The CRBO (100 µL) of different concentrations (0–12.5%) were added to each well and incubated for 24 h.

In the analysis, 100 µL of the MTT reagent (0.5 mg/mL) was added to each well and incubated for 2 h in the dark. The supernatant was removed from the well, and 100 µL dimethyl sulfoxide (DMSO) was added to dissolve the formazan crystals and the absorbance measured at 570 nm using a microplate reader (CLARIOstar^®^ Plus, BMG Labtech; Ortenberg, Germany). The results were expressed as a percentage of cell viability calculated by Equation (2). The concentration of CRBO to inhibit 50% cell viability (IC_50_) was also determined.

Cell viability (%) = (absorbance of the treated cell)/(absorbance of control cell) × 100(2)

#### 2.10.2. Intracellular Reactive Oxygen Species (ROS) Scavenging Capacity

The intracellular ROS scavenging capacity was assessed following the evaluation by Hamzeh et al. [10] with some modifications. HepG2 cells were seeded at a density of 7 × 10^3^ cells/well in a 96-well microplate containing 100 µL culture medium and incubated with 5% CO_2_ at 37 °C for 24 h. The cells were then treated with 100 µL of DMEM (without FBS and antibiotic) containing various concentrations of CRBO (0, 0.1, 0.2, and 0.39%) and incubated for 24 h, given that the CRBO concentrations of HE oil above 0.39% were cytotoxic. The positive control was 10 mM N-acetyl-L-cysteine (NAC) dissolved in 100 µL of DMEM. After incubation, the supernatant was discarded. The cells were further treated with 100 µL DMEM (i.e., control) or 100 µL DMEM containing 1 mM H_2_O_2_ to stimulate free radicals (oxidative stress) for 30 min, and the supernatant was then discarded. Afterward, 2’-7’dichlorofluorescin diacetate (DCFH-DA) was added, and the fluorescence intensity was measured at the excitation and emission wavelengths of 485 and 570 nm, respectively, using a microplate reader (CLARIOstar^®^ Plus, BMG Labtech; Ortenberg, Germany). The results were expressed as fold of relative 2′-7′dichlorofluorescin (DCF) fluorescence intensity over the control (i.e., the cells subjected to DMEM only).

### 2.11. Synchrotron-Radiation-Based Fourier Transform Infrared (SR-FTIR)

The SR-FTIR spectra of CRBO were measured at the Synchrotron Light Research Institute, Thailand. The CRBO was placed on the IR microscope (Hyperion 2000, Bruker Optics; Billerica, MA, USA) connected to FTIR spectrometer (Vertex 70, Bruker Optics; Billerica, MA, USA) equipped with the nitrogen-cooled mercury cadmium telluride (MCT) detector. The IR absorption spectra were scanned with the wavenumber of 4000 to 700 cm^−1^ at 64 scans and 4 cm^−1^ resolution. The OPUS 7.2 software was used to control the instrument and collect the FTIR spectra. The data preprocessing and analysis of SR-FTIR spectra followed Kongmon et al. [32].

### 2.12. Statistical Analysis

The results were expressed as the means ± standard deviations. The statistical analysis was carried out using Minitab^®^ 17 (Minitab Inc.; State College, PA, USA). One-way analysis of variance (ANOVA) and Tukey HSD comparison were used to determine the statistical difference, given the 5% significance level (*p* < 0.05).

## 3. Results and Discussion

### 3.1. Physicochemical Properties of CRBO

Table 1 tabulates the oil yield, free fatty acid (FFA), peroxide value (PV), ρ-anisidine value (ρ-AV), total oxidation value (TOTOX), and Lovibond color value of CRBO under different extraction processes (HE, CE, CCE, and UCE). The Soxhlet extraction using hexane as solvent (HE method) achieved the highest oil yield (18.41 g oil/100 g RB), consistent with [5,24]. The oil yields of CE, CCE, and UCE were between 14.27–17.31 g oil/100 g RB. The results showed that the extraction methods significantly affected the oil yields (*p* < 0.05). Thermal cooking (100 °C, 5 min) and ultrasound pretreatment (4.5 W/g, 10 min) followed by cold press extraction (CCE and UCE) significantly improved the oil yield, vis-à-vis the CE process. The higher oil yield of CCE and UCE could be attributed to disruption of cell walls by thermal cooking and ultrasound, resulting in improved liquid mass transfer [25,33,34].

FFA, PV, ρ-AV, and TOTOX are commonly used to evaluate the oil quality and processing and storage stability. The FFA value is the quantity of free fatty acids formed by the hydrolysis of oils, while PV indicates the levels of peroxides and hydroperoxides, which are the primary oil oxidation products formed during the early stages of oxidation [16]. The ρ-AV measures the levels of the secondary oil oxidation products produced during the oxidative degradation of oils, primarily aldehydes (such as 2-alkenals and 2,4-alka-dienals), leading to oil rancidity [15,35]. TOTOX is used to measure the hydroperoxides and oil oxidation products, indicating the progressive oxidative deterioration of oils [6].

In this research, FFA, PV, ρ-AV, and TOTOX were in the range of 3.31–4.97%, 5.09–8.33 meq O_2_/kg oil, 0.19–0.31, and 10.38–16.97, respectively (Table 1). The FFA, PV, ρ-AV, and TOTOX of CE and CCE oils were significantly lower than those of HE and UCE oils (*p* < 0.05), indicating the effect of extraction processes on the oil quality and oxidative stability. The higher FFA, PV, ρ-AV, and TOTOX of HE and UCE oils could be attributed to the degradation of lipids under high temperatures and ultrasound irradiation during extraction [6,24]. According to Bhatnagar et al. [14], Maszewska et al. [13], and Aksoylu-Özbek and Ergönül [35], high levels of PV and ρ-AV could be attributed to high prooxidants (chlorophylls and carotenoids). The levels of antioxidants (tocopherols and phenolics) influence the levels of PV and ρ-AV since tocopherols and phenolics could inactivate the formation of hydroperoxides.

In this research, the FFA levels in CRBO (3.31–4.97%) were comparable to Thanonkaew et al. [25] (3.17–5.58% for CBRO) and to Alfaro et al. [36] (3.23% for brown CRBO) but lower than Rajam et al. [37] and Van Hoed et al. [4] (7.85% and 9.98%, respectively, for CRBO). According to Punia et al. [26], Charoonratana [38], and Lavanya et al. [39], oils containing more than 5% FFA are unfit for human consumption. The PV levels (5.09–8.33 meq O_2_/kg oil) were lower than those in Thanonkaew et al. [25] (11.72–18.85 meq O_2_/kg oil). The ρ-AVs varied between 0.19 and 0.31, which were lower than in Maszewska et al. [13], who reported a ρ-AV of fresh RBO of 4.5.

The TOTOX values of HE, CE, CCE, and UCE were 16.32, 10.38, 10.41, and 16.97, respectively. The higher TOTOX values of HE and UCE oils indicated that the HE and UCE oils were more prone to oxidation degradation, unlike the CE and CCE oils which were less susceptible to oxidative deterioration due to lower TOTOX values. According to Maszewska et al. [13], a high TOTOX value indicates a high degree of oxidation. In addition, PV, ρ-AV, and TOTOX below 10 meq O_2_/kg oil, 8, and 10, respectively, are indicative of high-quality oils [13]. In this research, the lower FFA, PV, ρ-AV, and TOTOX of CE and CCE oils indicate the lower levels of oxidation products, resulting in higher oxidative stability and longer shelf life, vis-à-vis the HE and UCE oils.

Color is another important characteristic for evaluating the oil quality. The Lovibond color unit was measured by the Lovibond tintometer in red and yellow terms [40]. In this research, the Lovibond red and yellow values of CRBO were 1.00–1.50 and 5.40–7.40, respectively, and the Lovibond color (5R + Y) units of CRBO were 10.40–15.00 (Table 1). The Lovibond red, yellow, and 5R + Y units of HE and UCE oils were significantly higher than those of CE and CCE (*p* < 0.05). The higher Lovibond color values of HE and UCE oils could be attributed to Maillard products.

According to Phan et al. [24], thermal pretreatment in rice bran oil extraction caused the color of CRBO to turn dark due to the decomposition of chlorophylls into pheophytins and the facilitation of the extraction of pigments and impurities into the oil. In addition, Maillard products contributed to the redness of CRBO under high temperatures. According to Van Hoed et al. [4] and Zhou et al. [40], Maillard products played a more important role in the Lovibond color values of RBO than β-carotene and chlorophyll. In this research, the Lovibond color values (1.0–1.5 for R and 10.4–15.0 for 5R + Y) were comparable to Rajam et al. [37] (1–3 for R and 10–12 for 5R + Y for refined RBO) and to Rekha et al. [41] (13–24 for 5R + Y). According to Fengxia et al. [42] and Mingyai et al. [5], the Lovibond red, yellow, and 5R + Y units of most edible oils should be below 2.0, 10.0, and 20.0, respectively. Based on the Lovibond color unit, the quality of CE and CCE oils were better than that of HE and UCE oils.

### 3.2. Bioactive Compounds and Chemical Antioxidant Activity of CRBO

Rice bran oil is rich in bioactive compounds that improve oil oxidative stability and antioxidant capacity [37,38]. Table 2 tabulates the bioactive compounds and chemical antioxidant activity of CRBO. The bioactive compounds, including γ-oryzanol, α-tocopherol, TPC, and TFC, of UCE oil were significantly lower than those of HE, CE, and CCE oils (*p* < 0.05), demonstrating the effect of extraction processes on the bioactive compounds and quality of extracted oil. The lower bioactive compounds of UCE oil could be attributed to the ultrasound inducing peroxide radicals in the cavitation bubbles, resulting in accelerated chemical reactions and decomposition of lipids [24,43].

In Table 2, the γ-oryzanol contents of CRBO were 0.99–1.83 g/100 g, which were comparable to the findings of Pandey and Shrivastava [23] and Liu et al. [2] of 1.52 and 1.49 g/100 g of CRBO, respectively. However, the γ-oryzanol values were higher than in Mingyai et al. [44] and Alfaro et al. [36] (0.29–0.33 g/100 g). According to CODEX STAN 210 [45], Rajam et al. [37], Charoonratana [38], and Xu et al. [1], the γ-oryzanol of CRBO should be in the range of 0.9–2.9%, depending on the origin of the rice bran.

The α-tocopherol content of CRBO varied between 0.23 and 0.51 mg/g. The results were comparable to that of Soxhlet extraction and aqueous enzymatic extraction of rice bran (0.26 and 0.32 mg/g, respectively) [1] but higher than that of Alfaro et al. [36] and Rajam et al. [37] (0.11–0.13 mg/g for CRBO). According to Bakota et al. [46], CRBO is rich in α-tocopherol, varying between 0.049–0.583 mg/g.

In this study, TPC and TFC of CRBO varied between 3.79 and 6.57 mg GAE/g and 0.35 and 1.95 mg CE/g, respectively. Trevisani Juchen et al. [22] reported the TPC of parboiled rice bran oils from Soxhlet hexane and ethanol extraction of 0.80 and 1.65 mg GAE/g, respectively. Ali et al. [47] reported that the TPC and TFC of BRRI dhan 49 crude rice bran oil were 22.23 mg GAE/g and 8.67 mg CE/g. The discrepancies could be attributed to the origin of the rice bran and different extraction methods.

As shown in Table 2, ultrasound pretreatment adversely affected the bioactive phytochemicals (γ-oryzanol, α-tocopherol, TPC, and TFC) in UCE oil, particularly TFC. The ultrasound generated high pressure and high temperatures in the medium. However, the heat stability of bioactive phytochemicals is varied, resulting in different disintegration of the chemical structures. According to Chaaban et al. [48], flavonoids are heat sensitive, as evidenced by rapid degradation of flavonoids for temperatures above 100 °C. The HE, CE, and CCE oils contain more natural antioxidants than the UCE oil. However, cold press extraction (CE and CCE) is more environmentally friendly than the HE process, which requires hexane solvent. Essentially, CCE is most operationally and environmentally suitable for rice bran oil extraction.

The DPPH, ABTS, and FRAP assays are commonly used to determine the in vitro (chemical) antioxidant activity. Specifically, the DPPH and ABTS assays measure the activity of scavenging stable DPPH• and ABTS•+ radicals, while FRAP measures the activity of reducing ferric ions (Fe^3+^) to ferrous ions (Fe^2+^).

Table 2 tabulates the chemical antioxidant activity of CRBO from different extraction methods, including DPPH (2.95–6.21 mg TEAC/g), ABTS (4.97–10.35 mg TEAC/g), and FRAP (2.40–3.75 mg TEAC/g). The discrepancies in the antioxidant activity could be attributed to different mechanisms by which the assay methods were applied and to the properties of antioxidants [2,8]. The HE, CE, and CCE oils possessed higher antioxidant activity than the UCE oil, corresponding to the higher levels of bioactive compounds in HE, CE, and CCE oils, in comparison with the UCE oil. The higher radical scavenging activity could be attributed to the presence of natural antioxidants [1]. On the other hand, the UCE oil exhibited lower antioxidant activity due to ultrasound-induced peroxide radicals, subsequently degrading the natural antioxidants and reducing the DPPH and ABTS radical scavenging and ferric reducing activities [24]. Essentially, the HE, CE, and CCE oils contained high levels of natural antioxidants, contributing to enhanced radical scavenging and ferric ion reducing activities.

The antioxidant activity (DPPH, ABTS, and FRAB) and bioactive compounds (γ-oryzanol, α-tocopherol, TPC, and TFC) were significantly positively correlated (*p* < 0.001) (Table 3). The correlation between DPPH, ABTS, and FRAP were positively high (0.984–0.994), consistent with Dudonne et al. [9]. The correlation between TPC and the antioxidant activity was highest (0.986–0.998), followed by γ-oryzanol (0.975–0.992) and α-tocopherol (0.963–0.986). The bioactive compounds contributed to the chemical antioxidant activity of CRBO, consistent with Trevisani Juchen et al. [22] and Liu et al. [2].

### 3.3. Cellular Antioxidant Activity of CRBO

#### 3.3.1. Cell Viability Assessment

**Figure 1:**
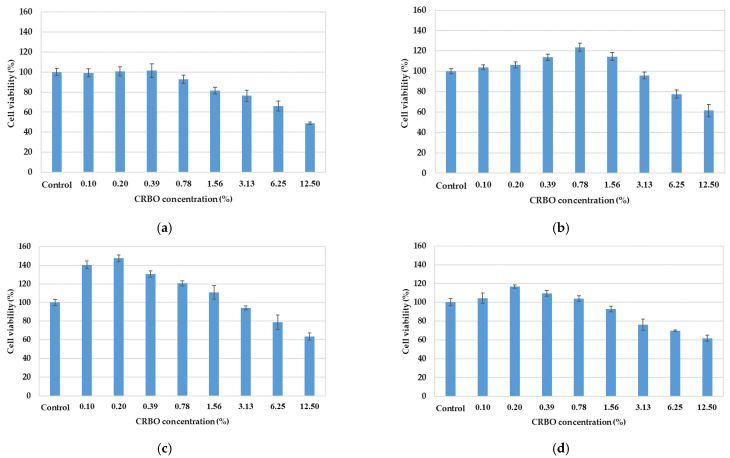
Cytotoxicity of variable concentrations of CRBO to HepG2 cells using MTT assay, where control is 0% CRBO concentration: (**a**) hexane extraction (HE); (**b**) cold press extraction (CE); (**c**) thermally pretreated cold press extraction (CCE); (**d**) ultrasound-pretreated cold press extraction (UCE). The values are the mean of three replications ± standard deviation.

Cellular antioxidant activity assay is used to assess the in vivo (cellular) antioxidant activity due to its ability to estimate the uptake and distribution of oil in the cells [2,10]. Figure 1 shows the cytotoxicity of CRBO from different extraction processes (HE, CE, CCE, UCE) to HepG2 cells, given the CRBO concentrations of 0–12.50% (0–125 mg/mL) where 0% CRBO was the control. The concentrations of CE and CCE oils below 1.56% exhibited no inhibitory effect on cell viability, with the respective IC_50_ of 16.45% and 17.26%. Meanwhile, the concentrations of HE and UCE oils below 0.39% and 0.78%, respectively, were noncytotoxic, with respective IC_50_ values of 12.24% and 16.42%. The results indicated that the HE and UCE oils were more cytotoxic to HepG2 cells than the CE and CCE oils. Specifically, the HE oil was most cytotoxic to HepG2 cells, followed by the UCE, CE, and CCE oils. The non-cytotoxic CRBO concentrations (0–0.39%) were used for the subsequent cellular antioxidant experiment.

#### 3.3.2. Intracellular Reactive Oxygen Species (ROS) Scavenging Capacity

**Figure 2:**
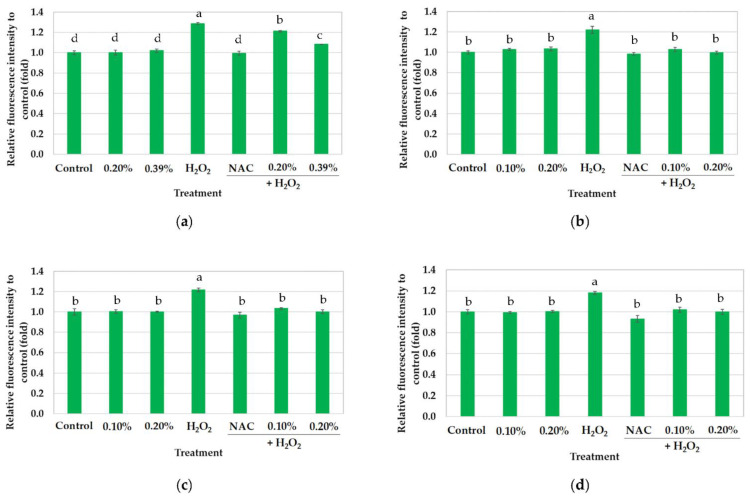
Intracellular ROS scavenging capacity under variable CRBO concentrations (0–0.39%). The results are expressed as fold of DCF fluorescence over the control, where control is cells treated with 100 µL DMEM without H_2_O_2_: (**a**) hexane extraction (HE); (**b**) cold press extraction (CE); (**c**) thermally pretreated cold press extraction (CCE); (**d**) ultrasound-pretreated cold press extraction (UCE). The values are the mean of three replications ± standard deviation. Different superscripts on bars indicate statistical differences between treatments (*p* < 0.05).

Figure 2 shows the intracellular ROS scavenging capacity of CRBO in fold of relative DCF fluorescence intensity over the control (i.e., the cells subjected to DMEM only). The CE, CCE, and UCE oils significantly reduced H_2_O_2_-induced ROS production in a dose-dependent fashion. The CE, CCE, and UCE oils (at 0.10% concentration) significantly inhibited the ROS production when compared with H_2_O_2_ treatment (*p* < 0.05) with the efficiency comparable to the positive control (i.e., NAC), indicating good cell protection against oxidative stress. Meanwhile, the intracellular ROS scavenging capacity of HE oil (0.20–0.39%) was lower than that of CE, CCE, and UCE oils (0.10–0.20%) and NAC.

Although the HE oil achieved higher chemical antioxidant activity than the UCE oil (Table 2), the cellular antioxidant activity of UCE oil was higher than that of HE oil. This contradictory finding could be attributed to certain compounds in HE oil interfering with the cell-based antioxidant activity evaluation, resulting in a reduction in the cellular antioxidant activity. Lu et al. [7] reported that vegetable oils, despite similar triglycerides, exhibited different levels of cellular antioxidant activity due to different bioactive compounds in the oils, especially polyphenols. The chemical- and cell-based antioxidant activity of Herbaceous peony seed oil was subject to various bioactive compounds [49]. According to Yuwang et al. [12], the DPPH and ABTS radicals are not present in humans, while the FRAP assay is performed under acidic conditions (pH 3.6) that are different from human physiological pH (pH 7.0–7.4), contributing to the inconsistent chemical- and cellular-based antioxidant activity.

Essentially, the CE, CCE, and UCE oils exhibited higher inhibitory effects against H_2_O_2_-induced cellular oxidative stress than the HE oil (Figure 2). Given the inconsistency between the chemical- and cell-based antioxidant activity of CRBO, subsequent research would thus investigate and identify the compounds that affect the cellular antioxidant activity and contribute to the discrepancy.

### 3.4. Squalene, Sterols, and Triterpenoid in CRBO

**Figure 3:**
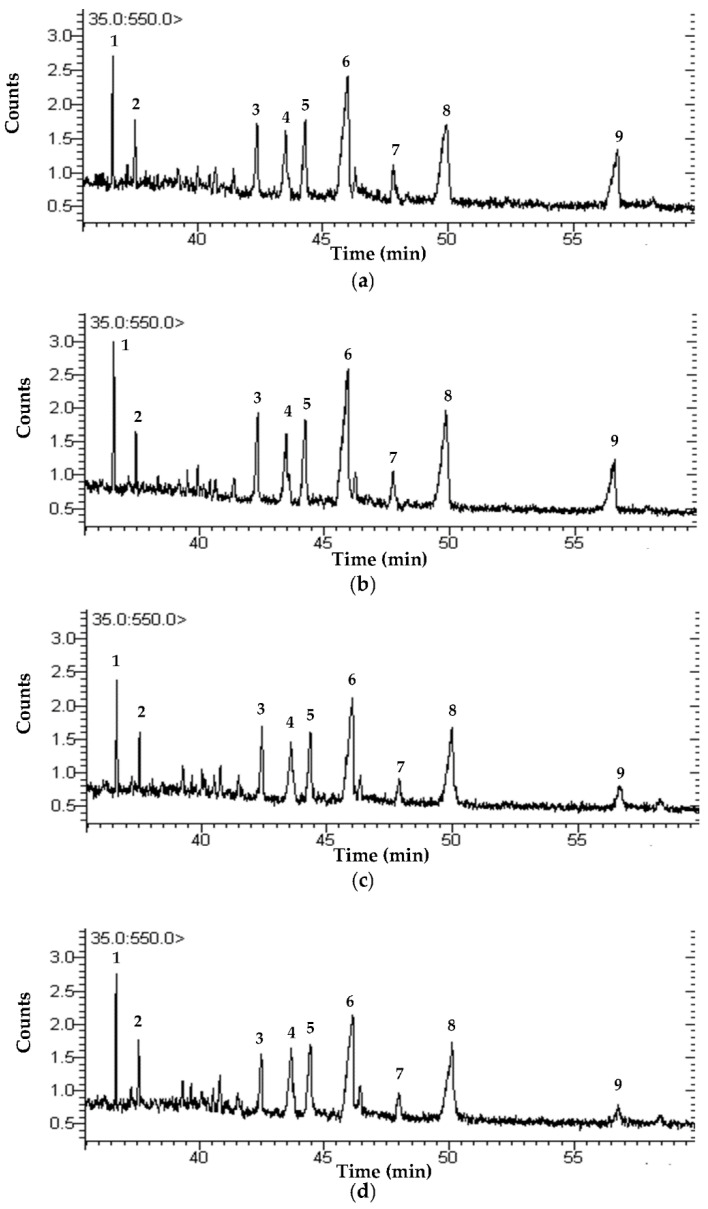
GC–MS chromatograms of CRBO from different extraction processes given the retention time of 35–60 min: (**a**) hexane extraction (HE); (**b**) cold press extraction (CE); (**c**) thermally pretreated cold press extraction (CCE); (**d**) ultrasound-pretreated cold press extraction (UCE). (**1**) squalene; (**2**) unidentified; (**3**) stigmasta-4,6,22-trien-3β-ol; (**4**) campesterol; (**5**) stigmasterol; (**6**) β-sitosterol; (**7**) cycloartenol acetate; (**8**) 24-methylenecycloartanol; and (**9**) propanoic acid, 3,3′-thiobis-, didodecyl ester.

Figure 3 shows the GC–MS chromatograms of phytochemicals (i.e., squalene, phytosterols, and triterpenoid) in CRBO from different extraction processes (HE, CE, CCE, UCE), given the retention time of 35–60 min. The characteristic peaks were similar for all CRBO. Table 4 tabulates the relative percentage composition of squalene and phytosterols (i.e., campesterol, stigmasterol, and β-sitosterol) and triterpenoid (24-methylenecycloartanol). Squalene is a precursor to sterol synthesis and possesses the antioxidant property [1,46,50]. According to Suttiarporn et al. [3], campesterol, stigmasterol, β-sitosterol, and 24-methylenecycloartanol possess various biological properties, including anti-inflammation and cytotoxicity to certain cancer cell lines.

The relative percentage composition of campesterol, stigmasterol, and β-sitosterol in CRBO were between 20.31–25.69%, 20.44–23.52%, and 50.79–59.22% of total phytosterols, depending on the oil types. The results were consistent with Shi et al. [51] and Xu et al. [1], who reported that β-sitosterol (42.9–53.8% of total phytosterols) was most abundant in vegetable oils, followed by campesterol (16.6–22.6%) and stigmasterol (10.4–14.1). Rajam et al. [37] reported that the phytosterols in CRBO were β-sitosterol (42.7%), campesterol (30.9%), and stigmasterol (26.4%). According to CODEX STAN 210 [45], β-sitosterol, campesterol, and stigmasterol in CRBO should be between 25.0 and 67.0%, 11.0 and 35.0%, and 6.0 and 40.0% of total phytosterols, respectively.

### 3.5. SR-FTIR Analysis of CRBO

**Figure 4:**
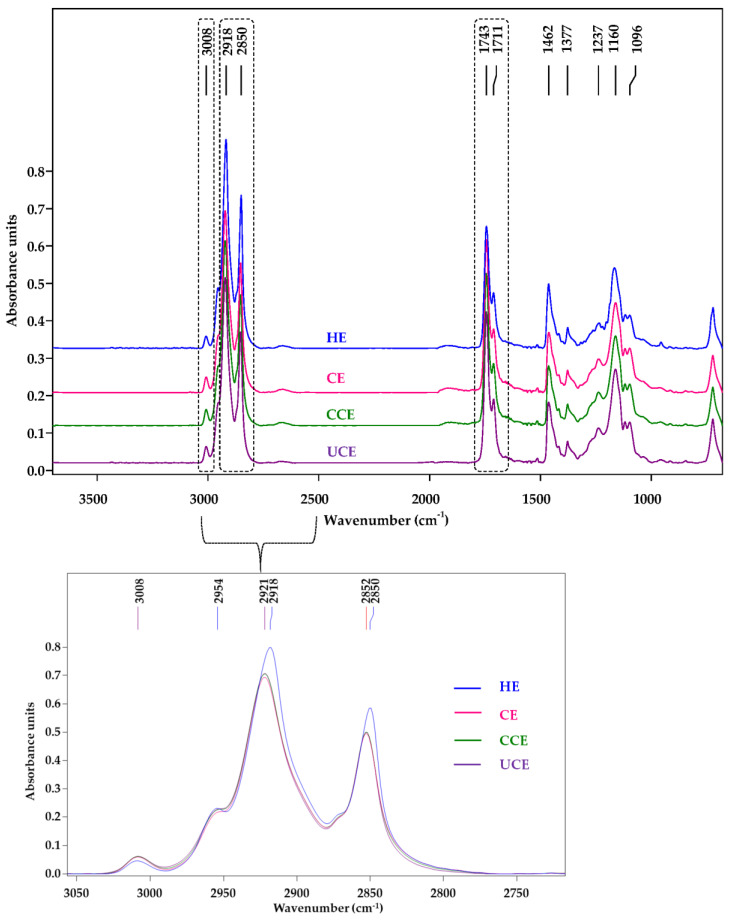
SR-FTIR spectra of CRBO from different extraction methods. HE: hexane extraction, CE: cold press extraction, CCE: thermally pretreated cold press extraction, UCE: ultrasound-pretreated cold press extraction.

In this research, SR-FTIR was used to evaluate the oxidative stability of CRBO from different extraction processes. The advantages of SR-FTIR over conventional FTIR include its detection of trace amounts, higher sensitivity, and signal-to-noise ratio [32]. Figure 4 shows the SR-FTIR spectra of CRBO for the spectral range of 3500–700 cm^−1^. The SR-FTIR spectra exhibited similar characteristic absorption peaks to those of conventional FTIR [15,18,20].

Table 5 tabulates the peak assignment that corresponds to specific vibration of the functional groups of CRBO. The absorption peak at 3008 cm^−1^ corresponds to the C–H stretching vibration of the cis-double bond. The peaks at 2918 and 2850 cm^−1^ belong to the C–H asymmetric and symmetric stretching vibration of the aliphatic CH_2_ group in triglycerides, respectively. The peak at 1743 cm^−1^ corresponds to C=O stretching of the ester group of triglycerides, while the peak at 1711 cm^−1^ belongs to C=O stretching of the acidic group of free fatty acids [15,17,20].

**Figure 5:**
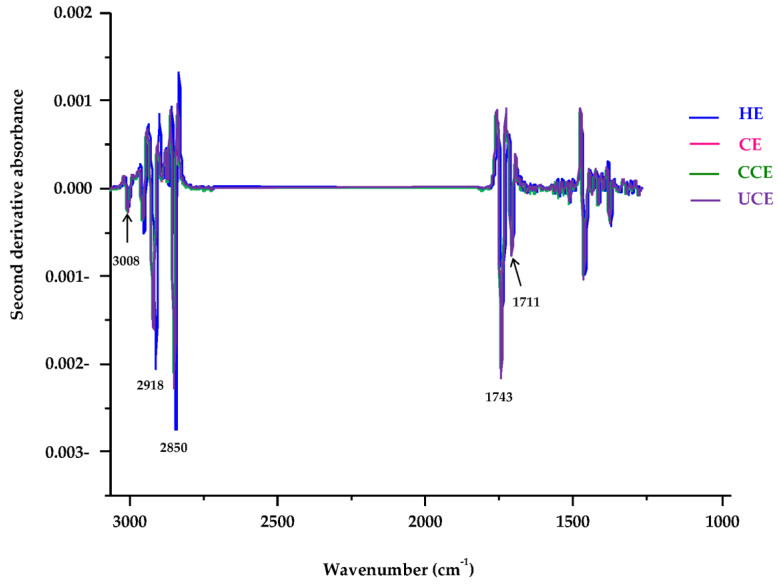
The second derivative SR-FTIR absorption spectra of CRBO in the 3050–1250 cm^−1^ region. HE: hexane extraction, CE: cold press extraction, CCE: thermally pretreated cold press extraction, UCE: ultrasound-pretreated cold press extraction.

In Figure 4, the absorption peak at 3008 cm^−1^ of HE oil was lowest, and the second and third peaks of HE oil were at 2918 and 2850 cm^−1^, vis-à-vis at 2921 and 2852 cm^−1^ for the CE, CCE, and UCE oils. To increase the specificity of absorption peaks, the spectra of the 3050–1250 cm^−1^ region were preprocessed with the Savitzky-Golay second derivative, as shown in Figure 5. The second derivative SR-FTIR absorption spectra demonstrated the difference between CRBO (i.e., HE, CE, CCE, UCE oils) for the peaks of 3008 to 2850 cm^−1^ and 1743 to 1711 cm^−1^. The reduction in peak intensity at 3008 cm^−1^ of HE oil could be attributed to decreases in cis unsaturated fatty acids [15,19].

**Figure 6:**
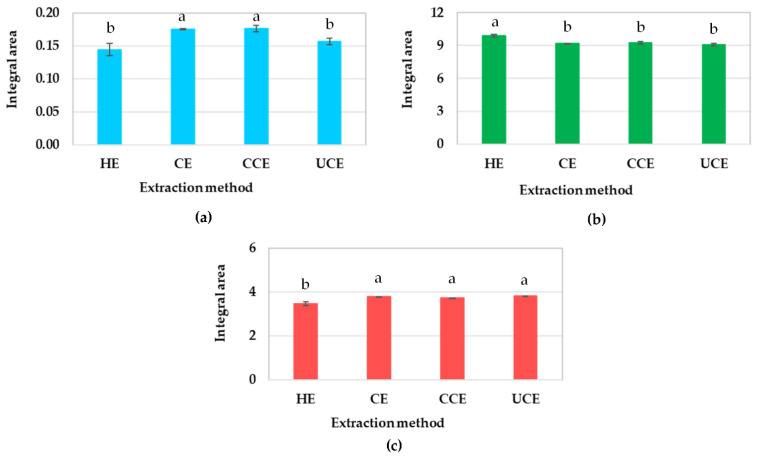
The integral areas of absorption peaks at: (**a**) 3008 cm^−1^; (**b**) 2918–2850 cm^−1^; (**c**) 1743–1711 cm^−1^. HE: hexane extraction, CE: cold press extraction, CCE: cooking process combined with cold press extraction, UCE: ultrasonic pretreatment combined with cold press extraction. The values are the mean of three replications ± standard deviation. Different superscripts on bars indicate statistical differences between extraction methods (*p* < 0.05).

Figure 6 shows the integral areas in which the intensity at 3008 cm^−1^ of HE and UCE oils were significantly lower than those of CE and CCE oils (*p* < 0.05). According to Rohman and Man [15], the FTIR absorbance at 3008 cm^−1^ could be used to characterize ρ-AV, given the high inverse correlation between the FTIR absorbance at 3008 cm^−1^ and ρ-AV (R^2^ = 0.844). In this research, the SR-FTIR intensities of CE and CCE oils at 3008 cm^−1^ (Figure 6a) were inversely associated with the ρ-AV values (0.2 and 0.19; Table 1). As a result, the CE and CCE oils possess higher oxidative stability than the HE and UCE oils because of lower ρ-AV, which in turn retards the oil oxidation degradation.

The distinct increase in peak intensity of HE oil was observed at 2918 and 2850 cm^−1^ and decreased at 1743 and 1711 cm^−1^ when compared to those of CE, CCE, and UCE oils (Figure 4, Figure 5 and Figure 6). According to Javed et al. [18], the absorption peaks at 2924 and 1710 cm^−1^ (2918 and 1711 cm^−1^ in this study) belonged to free fatty acids, including linoleic acid, oleic acid, palmitic acid, and stearic acid. Vlachos et al. [20] reported that the absorption peaks at 1746 and 1700 cm^−1^ (1743 and 1711 cm^−1^ in this study) were attributed to triglycerides and free fatty acids, respectively. The findings verify the effect of different extraction processes on the chemical composition of CRBO.

**Figure 7:**
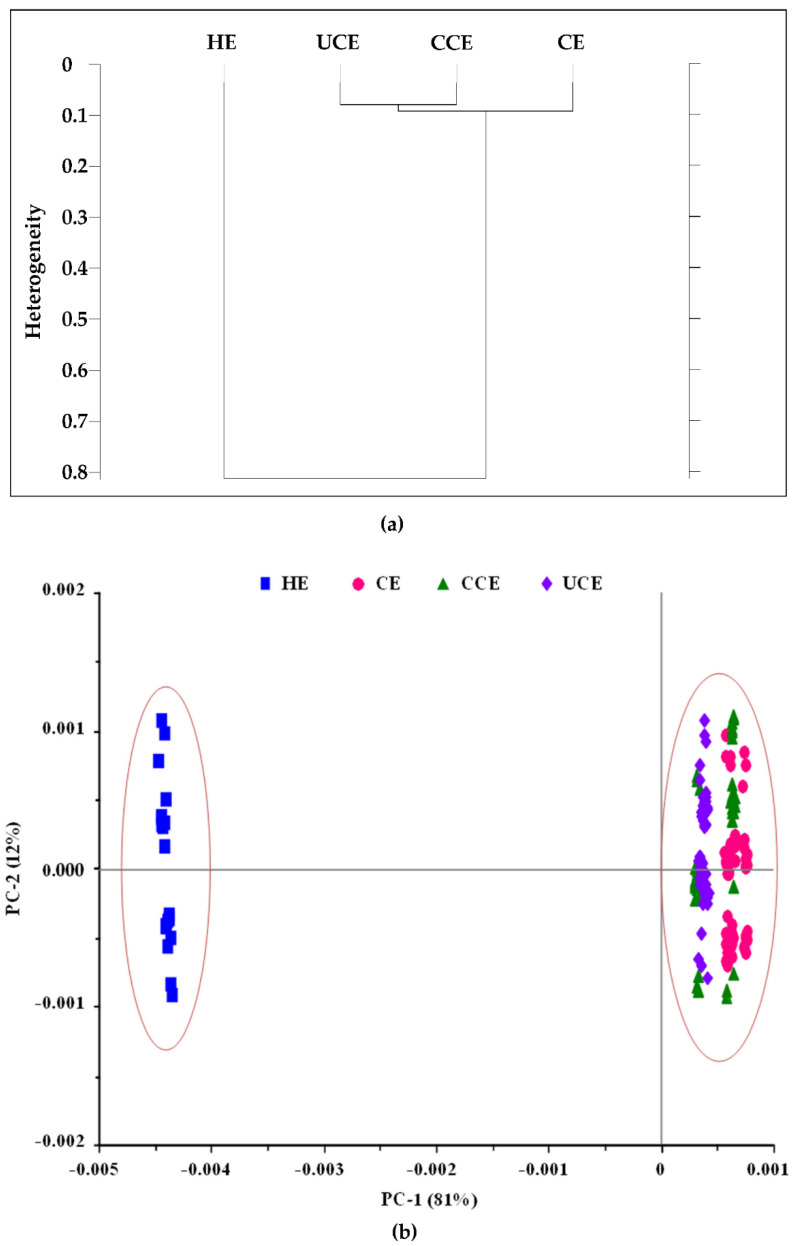
(**a**) Two-dimensional HCA dendrograms of CRBO from different extraction processes; (**b**) two-dimensional PCA score plot showing the first and second principal components (PC-1 and PC-2) for the SR-FTIR spectra.

Furthermore, hierarchical cluster analysis (HCA) and principal component analysis (PCA) were used to visualize the cluster pattern of CRBO based on the SR-FTIR spectra (Figure 7). The HCA was performed using the second derivative and vector normalized SR-FTIR spectra of HE, CE, CCE, and UCE oils in the spectral range of 3050–1250 cm^−1^. Figure 7a shows the two-dimensional HCA results in dendrograms, consisting of two clusters based on the differences in peak intensity at 3008, 2918, 2850, 1743, and 1711 cm^−1^: the CE, CCE, and UCE oils in the first cluster and the HE oil in the second cluster.

The PCA of the SR-FTIR spectra was performed to validate the HCA results. Figure 7b shows the 2D score plot of PCA based on the SR-FTIR spectra of CRBO. The first two principal components (PC) accounted for 93% of total data variation, with PC-1 accounting for 81% and PC-2 for 12% variance. The CRBO were categorized into two groups based on the differences in peak intensity at 3008, 2918, 2850, 1743, and 1711 cm^−1^, consisting of the CE, CCE, and UCE oils in the first group and the HE oil in the second group. In Figure 7b, the principal components of CCE oil overlaps with those of CE and UCE oils, suggesting that the chemical composition of CCE oil closely resembled that of CE and UCE oils. The results confirm that the extraction processes affect the chemical properties of CRBO.

Essentially, the HE process, despite high oil yield, achieves low oxidative stability and is chemically toxic. The CE oil possesses high oxidative stability and is eco-friendly but achieves low oil yield. The UCE process is eco-friendly with high oil yield, but the oil quality is lower than that of CE and CCE oils. In comparison, the CCE process is operationally and environmentally suitable for improving the oil extractability, oxidative stability, bioactive compounds, and antioxidant activities of CRBO. In addition to high-quality CRBO, the CCE process is simple, low-cost, and eco-friendly. As a result, the CCE method holds great potential for large-scale production of high-quality CRBO.

## 4. Conclusions

This research comparatively investigated the effects of extraction processes on the oxidative stability, bioactive phytochemicals, and chemical and cellular antioxidant activities of CRBO. The experimental extraction processes included HE, CE, CCE, and UCE. The results showed that thermal cooking and ultrasound pretreatment followed by cold press extraction (CCE and UCE) enhanced the oil extractability of CRBO. The oxidative stability of HE and UCE oils was lower than that of CE and CCE oils, as evident in the lower SR-FTIR integral areas of ρ-AV of HE and UCE oils. The Lovibond color units of CE and CCE oils were lower than those of HE and UCE oils, indicating the higher quality of CE and CCE oils. However, the bioactive compounds and chemical antioxidant activity of UCE oil were lowest as a result of the degradation of bioactive compounds during the ultrasound pretreatment. The cellular antioxidant assay indicated that the CE, CCE, and UCE oils were more potent in inhibiting H_2_O_2_-induced cellular oxidative stress, vis-à-vis the HE oil. The research findings demonstrated that thermally pretreated cold press extraction (CCE) is operationally and environmentally suitable for improving the oil yield, oxidative stability, bioactive compounds, and antioxidant activities of CRBO. Essentially, the CCE method holds great potential for large-scale production of high-quality CRBO. However, subsequent research would investigate and identify the compounds that affect the cellular antioxidant activity and contribute to the discrepancy between the chemical- and cell-based antioxidant activities of CRBO.

## Figures and Tables

**Table 1:** Extraction yields and physicochemical properties of CRBO from different extraction processes.

Extraction Method	Yield (g Oil/100 g RB)	FFA (% Oleic acid)	PV (Meq O_2_/kg Oil)	ρ-AV	TOTOX	Lovibond Color Value		
						Red (R)	Yellow (Y)	5R + Y
HE	18.41 ± 0.50 ^a^	4.94 ± 0.11 ^a^	8.01 ± 0.10 ^a^	0.30 ± 0.01 ^a^	16.32 ± 0.05 ^a^	1.50 ± 0.02 ^a^	7.50 ± 0.01 ^a^	15.00 ± 0.05 ^a^
CE	14.27 ± 0.56 ^c^	3.31 ± 0.11 ^b^	5.09 ± 0.17 ^b^	0.20 ± 0.01 ^b^	10.38 ± 0.07 ^b^	1.00 ± 0.02 ^c^	5.40 ± 0.01 ^c^	10.40 ± 0.05 ^d^
CCE	17.31 ± 0.23 ^b^	3.53 ± 0.14 ^b^	5.11 ± 0.12 ^b^	0.19 ± 0.01 ^b^	10.41 ± 0.05 ^b^	1.00 ± 0.01 ^c^	5.50 ± 0.02 ^c^	11.00 ± 0.06 ^c^
UCE	16.68 ± 0.71 ^b^	4.97 ± 0.15 ^a^	8.33 ± 0.11 ^a^	0.31 ± 0.01 ^a^	16.97 ± 0.05 ^a^	1.25 ± 0.02 ^b^	6.50 ± 0.02 ^b^	12.75 ± 0.05 ^b^

HE: hexane extraction, CE: cold press extraction, CCE: thermally pretreated cold press extraction, UCE: ultrasound-pretreated cold press extraction. FFA: free fatty acid, PV: peroxide value, ρ-AV: ρ-anisidine value. The values are the mean of three replications ± standard deviation. Different superscripts in each column indicate statistical differences between extraction methods (*p* < 0.05).

**Table 2:** Bioactive phytochemicals and chemical antioxidant activity of CRBO from different extraction processes.

Extraction Method	γ-Oryzanol (g/100 g)	α-Tocopherol (mg/g)	TPC (mg GAE/g)	TFC (mg CE/g)	DPPH (mg TEAC/g)	ABTS (mg TEAC/g)	FRAP (mg TEAC/g)
HE	1.83 ± 0.04 ^a^	0.51 ± 0.03 ^a^	6.51 ± 0.25 ^a^	1.95 ± 0.02 ^a^	5.94 ± 0.28 ^a^	9.38 ± 0.15 ^b^	3.55 ± 0.15 ^ab^
CE	1.80 ± 0.05 ^ab^	0.48 ± 0.03 ^ab^	6.57 ± 0.21 ^a^	1.94 ± 0.02 ^a^	6.21 ± 0.28 ^a^	10.35 ± 0.15 ^a^	3.75 ± 0.11 ^a^
CCE	1.82 ± 0.05 ^a^	0.50 ± 0.02 ^a^	6.43 ± 0.12 ^ab^	1.94 ± 0.05 ^a^	5.97 ± 0.25 ^a^	9.93 ± 0.17 ^ab^	3.50 ± 0.10 ^ab^
UCE	0.99 ± 0.02 ^c^	0.23 ± 0.01 ^c^	3.79 ± 0.15 ^c^	0.35 ± 0.01 ^b^	2.95 ± 0.14 ^b^	4.97 ± 0.19 ^c^	2.40 ± 0.09 ^b^

HE: hexane extraction, CE: cold press extraction, CCE: thermally pretreated cold press extraction, UCE: ultrasound-pretreated cold press extraction. The values are the mean of three replications ± standard deviation. Different superscripts in each column indicate statistical differences between extraction methods (*p* < 0.05).

**Table 3:** Pearson correlation coefficients between bioactive phytochemicals and chemical antioxidant activity of CRBO.

Correlation	γ-Oryzanol	α-Tocopherol	TPC	TFC	DPPH	ABTS
α-Tocopherol	0.994 *					
TPC	0.996 *	0.992 *				
TFC	0.998 *	0.986 *	0.994 *			
DPPH	0.992 *	0.986 *	0.998 *	0.991 *		
ABTS	0.981 *	0.963 *	0.986 *	0.986 *	0.991 *	
FRAP	0.975 *	0.967 *	0.989 *	0.973 *	0.994 *	0.984 *

* denotes the statistical significance at 0.1% (*p* < 0.001).

**Table 4:** GC–MS relative percentage composition of squalene, phytosterols, and triterpenoid in CRBO.

Extraction Method	Squalene (% in CRBO)	Phytosterols (% in CRBO, % Total Phytosterols)			24-Methylenecycloartanol (% in CRBO)
		Campesterol	Stigmasterol	β-Sitosterol	
HE	0.14	0.25, 20.31	0.25, 20.47	0.73, 59.22	0.57
CE	0.15	0.28, 20.83	0.28, 21.36	0.76, 57.80	0.63
CCE	0.18	0.36, 25.69	0.33, 23.52	0.70, 50.79	0.63
UCE	0.15	0.32, 25.54	0.28, 22.44	0.66, 52.02	0.54

HE: hexane extraction, CE: cold press extraction, CCE: thermally pretreated cold press extraction, UCE: ultrasound-pretreated cold press extraction.

**Table 5:** Peak assignment for CRBO from different extraction processes.

Wavenumber (cm^−1^)	Type of Vibration and Functional Group Assignment
3008	C–H stretching of the *cis*-double bond (C=CH)
2918, 2921	C–H asymmetric stretching of the aliphatic CH_2_ group in triglycerides
2850, 2852	C–H symmetric stretching of the aliphatic CH_2_ group in triglycerides
1743	C=O stretching of the carbonyl group in ester groups of triglycerides
1711	C=O stretching of the carbonyl group in acidic groups of free fatty acids
1462	C–H bending (scissoring) of the aliphatic CH_2_ and CH_3_ groups
1377	C–H symmetric bending of the CH_3_ group
1235, 1237	C–O stretching of the ester group
1158, 1160	C–O stretching of the ester group
1096	C–O stretching

## Data Availability

The data presented in this study are available on request from the corresponding author.
